# Three-Dimensional Printing of Cellulose/Covalent Organic Frameworks (CelloCOFs) for CO_2_ Adsorption and Water Treatment

**DOI:** 10.1021/acsami.3c13966

**Published:** 2023-12-14

**Authors:** Hani Nasser Abdelhamid, Sahar Sultan, Aji P. Mathew

**Affiliations:** † Division of Materials and Environmental Chemistry, Stockholm University, Svante Arrhenius väg 16 C, Stockholm SE-10691, Sweden; ‡ Department of Chemistry, Faculty of Science, Assiut University, Assiut 71515, Egypt; § Nanotechnology Research Centre (NTRC), The British University in Egypt (BUE), Suez Desert Road, P.O. Box 43, El-Shorouk City 11837, Cairo, Egypt; ∥ Wallenberg Wood Science Center, Teknikringen 56-58, Stockholm 100 44, Sweden

**Keywords:** covalent organic frameworks, 3D printing, CO_2_ adsorption, water treatment

## Abstract

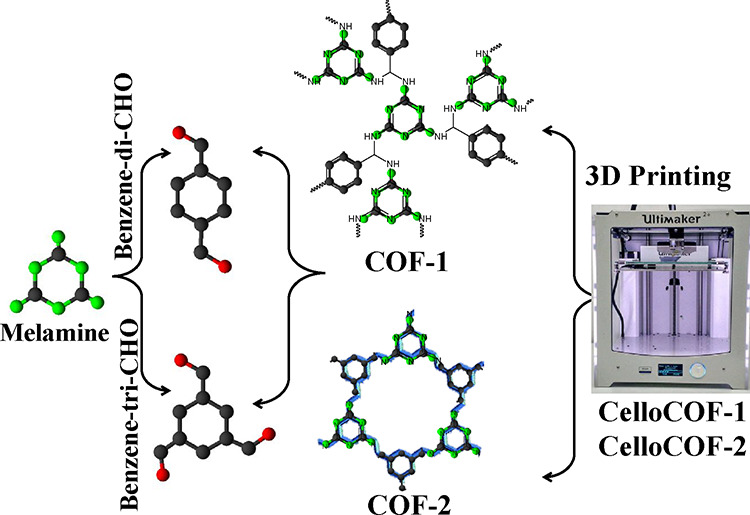

The development of porous organic polymers, specifically covalent organic frameworks (COFs), has facilitated the advancement of numerous applications. Nevertheless, the limited availability of COFs solely in powder form imposes constraints on their potential applications. Furthermore, it is worth noting that COFs tend to undergo aggregation, leading to a decrease in the number of active sites available within the material. This work presents a comprehensive methodology for the transformation of a COF into three-dimensional (3D) scaffolds using the technique of 3D printing. As part of the 3D printing process, a composite material called CelloCOF was created by combining cellulose nanofibrils (CNF), sodium alginate, and COF materials (i.e., COF-1 and COF-2). The intervention successfully mitigated the agglomeration of the COF nanoparticles, resulting in the creation of abundant active sites that can be effectively utilized for adsorption purposes. The method of 3D printing can be described as a simple and basic procedure that can be adapted to accommodate hierarchical porous materials with distinct micro- and macropore regimes. This technology demonstrates versatility in its use across a range of COF materials. The adsorption capacities of 3D CelloCOF materials were evaluated for three different adsorbates: carbon dioxide (CO_2_), heavy metal ions, and perfluorooctanesulfonic acid (PFOS). The results showed that the materials exhibited adsorption capabilities of 19.9, 7.4–34, and 118.5–410.8 mg/g for CO_2_, PFOS, and heavy metals, respectively. The adsorption properties of the material were found to be outstanding, exhibiting a high degree of recyclability and exceptional selectivity. Based on our research findings, it is conceivable that the utilization of custom-designed composites based on COFs could present new opportunities in the realm of water and air purification.

## Introduction

Covalent organic frameworks (COFs) have been recognized as highly prospective materials for a diverse array of applications encompassing gas storage, separation, catalysis, energy storage, and biological applications.^1−4^ The characteristics of these materials were considered advantageous because of their intrinsic capacity to adapt to synthetic conditions, remarkable resistance to chemical degradation, and the ability to change their porosity while maintaining a significant surface area. However, these materials demonstrate an increased tendency for aggregation. Three-dimensional (3D) printing has demonstrated potential among the several methodologies outlined for the processing of COFs.^5−9^ The utilization of 3D printing technology facilitates the creation of a hierarchical porous structure that possesses tailored features. The utilization of Pluronic F127^8,10^ and graphene oxide^11^ in the context of 3D printing for COFs was reported. The fabrication process of imine COFs via 3D printing necessitated the utilization of a 3D printing template and the execution of multiple sequential procedures.^10^ Certain additives, such as Pluronic F127, were identified to possess characteristics that are detrimental to the environment.^8,10^ Biopolymers, such as cellulose^12,13^ or sodium alginate,^14^ demonstrate favorable characteristics suitable for 3D printing applications. They facilitate the process of additive manufacturing for the production of multiple types of materials. The 3D printing of COFs requires the incorporation of environmentally sustainable additions such as biopolymers.

Climate change poses a significant threat to the human population.^15^ Carbon dioxide (CO_2_) is responsible for approximately 60% of the overall contribution to global warming.^16^ The imperative to address climate change necessitates the implementation of adsorption and conversion techniques (such as reduction, photoreduction, and fixation) for the removal of CO_2_ from the atmosphere.^17,18^ Among the several materials that have been reported, COFs show promise for the removal of CO_2_ through the processes of adsorption and conversion. The utilization of COFs has been found to enhance both the selectivity and effectiveness of CO_2_ adsorption, as demonstrated in previous studies.^16,19^ In addition, these tools can also serve the purposes of fixation and conversion.^16,20^ The adsorption performance of materials can be enhanced through the customization of their structure.^21^ Nevertheless, the conventional form of COFs in powder presents challenges in terms of their practical application.

The provision of clean water for both drinking and agricultural use has emerged as a pressing concern in light of the presence of pollutants. Approximately 2 billion individuals are currently under water stress, as shown by a recent study.^22^ Numerous toxins, including both organic and inorganic substances, have been discharged into the aquatic environment, resulting in significant global water contamination. The primary chemical hazards of utmost concern are associated with inorganic elements, particularly heavy metals, as well as organic pollutants, such as per- and polyfluoroalkyl substances (PFASs), e.g., perfluorooctanesulfonate (PFOS) and perfluorooctanoic acid (PFOA). These substances were regarded as persistent organic pollutants.^23^ Heavy metals exhibit a lower degree of degradability in comparison to organic contaminants.^24^ Moreover, these substances have a significant degree of solubility in aqueous solutions, displaying a notable capacity for movement within the water medium. Various materials, such as COFs, have been utilized to effectively remove PFASs and ions, demonstrating strong adsorption capabilities.^25−29^

Natural biopolymers such as cellulose advanced in several applications including water treatment.^30,31^ TEMPO (2,2,6,6-tetramethylpiperidine-1-oxyl)-assisted cellulose nanofibers (TOCNFs) refer to a specific category of cellulose nanofibers that are manufactured by a chemical oxidation technique known as TEMPO oxidation.^32^ The aforementioned procedure induces a transformation of the cellulose surface, resulting in a significant increase in the negative charge. This augmented negative charge facilitates the repulsion between nanofibers, leading to their enhanced dispersion in aqueous solutions.^33^ TOCNFs provide several distinctive characteristics that render them highly appealing for a diverse range of applications.^34^ The high aspect ratio of TOCNFs is characterized by its length-to-width ratio, which can reach several hundred times longer than its width.^35^ This unique feature contributes to its exceptional surface area and remarkable strength. TOCNFs exhibit a high degree of crystallinity, indicating a well-organized arrangement of cellulose chains with close packing.^36^ This characteristic imparts significant strength and rigidity to the material.^35,37^ Transparency is a notable characteristic of TOCNFs, rendering them well-suited for applications that prioritize optical clarity.^38^ TOCNFs are biocompatible with living beings without causing harm.^30^ These characteristic features of TOCNFs render them appropriate for utilization in several contexts including water treatment.^39,40^

This study introduces a thorough methodology for the production of nanocomposite materials, cellulose/sodium alginate (SA)/COFs, through the utilization of 3D printing technology. The aforementioned materials are denoted as 3D CelloCOF. Cellulose derivatives were utilized in the form of oxidized cellulose nanofibrils that incorporated carboxylic functional groups. Two separate COFs, i.e., COF-1 and COF-2, were utilized in the processing of materials. The composite materials were produced through implementation of the direct ink writing (DIW) technique within a 3D printing procedure. The 3D CelloCOF materials have remarkable adsorption properties for CO_2_, heavy metals, and PFOS. The materials exhibited notable adsorption capabilities, attractive recyclability, significant selectivity, and speedy achievement of equilibrium.

## Experimental Section

### Materials and Methods

Melamine, terephthaldehyde, benzene-1,3,5-tricarboxaldehyde, TEMPO, sodium alginate (SA), calcium chloride (CaCl_2_), PFOS, zinc nitrate (Zn(NO_3_)_2_·6H_2_O), potassium chromate (K_2_CrO_4_), copper sulfate (CuSO_4_), cobalt nitrate (Co(NO_3_)_2_), sodium chloride (NaCl), ferric chloride (FeCl_3_), aluminum chloride (AlCl_3_), acetone, dimethyl sulfoxide (DMSO), and tetrahydrofuran (THF) were purchased from Sigma-Aldrich (Germany). TEMPO-oxidized cellulose nanofibril (TOCNF, 1 wt %) was prepared via the reported method in ref (41).

### Synthesis of COF-1 and COF-2

The synthesis of COF-1 involves the mixing of melamine (0.313 g) and terephthalaldehyde (0.250 g) into DMSO (15.5 mL) and heating for 72 h at 180 °C.^42^ The materials were separated via filtration. The product was washed several times using acetone and THF before drying under vacuum (120 °C). Following the same procedure, we synthesized COF-2 using benzene-1,3,5-tricarboxaldehyde instead of terephthalaldehyde.

### 3D Printing CelloCOF-1 and CelloCOF-2

The inks of COF-1 or COF-2 were prepared using TOCNF/SA. Typically, COFs (COF-1 or COF-2, 10 g) were mixed with TOCNF (5 g). The mixture was diluted to 500 mL and mixed via an UltraTurrax (IKA, Sweden) at 15,000 rpm (30 min). CelloCOF was separated via centrifugation (10,000 rpm, 30 min). CelloCOFs (28.2 g) were mixed with SA (4.22 g, 6 wt %) for 3D printing.

The ink was printed via a 3D printer (Ultimaker 2+, Ultimaker, Canada) using the Discov3ry paste printing system (Structur3D Printing). The printed objects were designed using a computer-aided design (CAD) model. The inks were extruded via a nozzle (410 μm). The printed materials were stored in a solution of CaCl_2_ (6 wt %). For characterization and application, the ink and 3D-printed objects were dried via a freeze-dryer (ALPHA 1–2 Ldplus, Germany).

### Characterization

The functional groups and interactions among the functional groups of TOCNF/COF and adsorption of pollutants were characterized using Fourier transform infrared (FT-IR, Varian 670-IR FTIR spectrometer, UK). Phase identification of the COFs was performed using X-ray diffraction (XRD, PANalytical X’Pert PRO MPD). Thermal stability was determined using thermogravimetric analysis (TGA, PerkinElmer TGA 7).

The porosity and surface area analysis were evaluated using nitrogen sorption isotherms (Micromeritics ASAP 2020 instrument, 77 K). The materials were degassed at 110 °C under reduced pressure (10 Pa) for 10 h. The Brunauer–Emmett–Teller (BET) model was used to calculate the specific surface area. The pore size distribution (PSD) was determined via nonlocal density functional theory (NLDFT) using a model of N_2_@77 K on carbon slit pores.

The materials were imaged by using transmission electron microscopy (TEM, JEM 2100, JEOL, Japan) and scanning electron microscopy (SEM, Hitachi TM-3000, Japan). The elemental analysis and mapping were evaluated via SEM with an accelerating voltage of 15 kV using the analysis model.

### CO_2_ Adsorption–Desorption

The adsorption–desorption isotherms of CO_2_ were evaluated using a Micromeritics ASAP 2020 instrument (77 K). The materials were degassed at a pressure, temperature, and time of <10 Pa, 110 °C, and 10 h, respectively. The processes of both adsorption and desorption were achieved at 0 °C. The recyclability of the process was performed using the same equipment without any pretreatment.

### Heavy Metal Adsorption

Stock solutions (1000 ppm) of metal ions (Cu^2+^, Co^2+^, Zn^2+^, Ca^2+^, Fe^3+^, Al^3+^, CrO_4_^2–^ , and Na^+^) were prepared in deionized water. The printed materials were cut into small pieces (100 mg) and soaked in the metal solution (100 mL) for 12 h. The materials were decanted and dried before metal analysis using EDX.

The effect of the initial concentration was tested for Cu^2+^ using 5, 10, 50, and 100 ppm following the same procedure. Meanwhile, the selectivity was measured using Cu^2+^, Ni^2+^, Zn^2+^, Co^2+^, Cd^2+^, Fe^3+^, Al^3+^, and CrO_4_^2–^. A stock solution was prepared by dissolving 0.5 g of each metal in 250 mL. A piece of the 3D printed objects was soaked in 10 mL of the stock solution.

Recyclability for heavy metal ions using copper ions (10 ppm) as a model was performed. After each adsorption run, the material was soaked in 0.1 M HCl for 3 h at room temperature. The material was then used for the next cycle by using the same procedure mentioned above. The concentration of Cu^2+^ ions was measured using inductively coupled plasma atomic emission spectroscopy (ICP-OES, Thermo Scientific iCAP 6200).

### Adsorption of PFOS

A stock solution of PFOS was prepared at a concentration of 1000 ppm. 3D CelloCOF (100 mg) was soaked in 100 mL of PFOS. The kinetics of the adsorption was determined by withdrawing 0.5 mL of the solution at different times. The samples collected were analyzed by using high-performance liquid chromatography–mass spectrometry (Shimadzu LC-2040C 3D HPLC). The adsorption and distribution of PFOS on the surface of 3D CelloCOF was evaluated using EDX mapping.

The selectivity of PFOS was measured using a mixture of metal ions, i.e., 5 mL of Cu^2+^, Ni^2+^, Co^2+^, Cd^2+^, Zn^2+^, Fe^3+^, Al^3+^, and CrO_4_^2–^ (1000 ppm) and PFOS (5 mg). A piece of the 3D printed objects was soaked in 10 mL of the stock solution for 3 h. The elemental species after adsorption were determined using ICP-OES. The adsorption of PFOS was determined in terms of sulfur present in the chemical composition. The selectivity toward different adsorbates was determined using the following equation
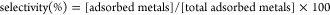


The selectivity of different adsorbents, i.e., CelloCOF-1 and CelloCOF-2, for each adsorbate was determined based on the adsorbed amount for each species using the following equation:
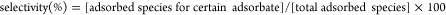


## Results and Discussion

### 3D Printing and Characterization

**Figure 1:**
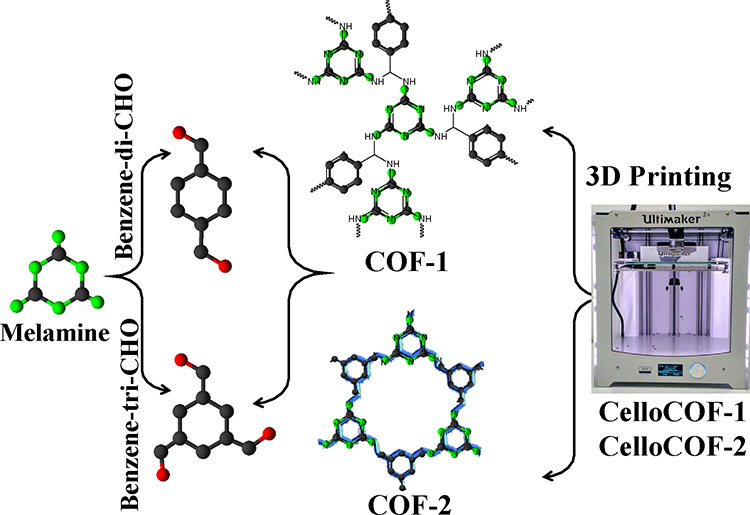
Schematic representation for synthesizing COF-1 and COF-2 for 3D printing into scaffolds.

**Figure 2:**
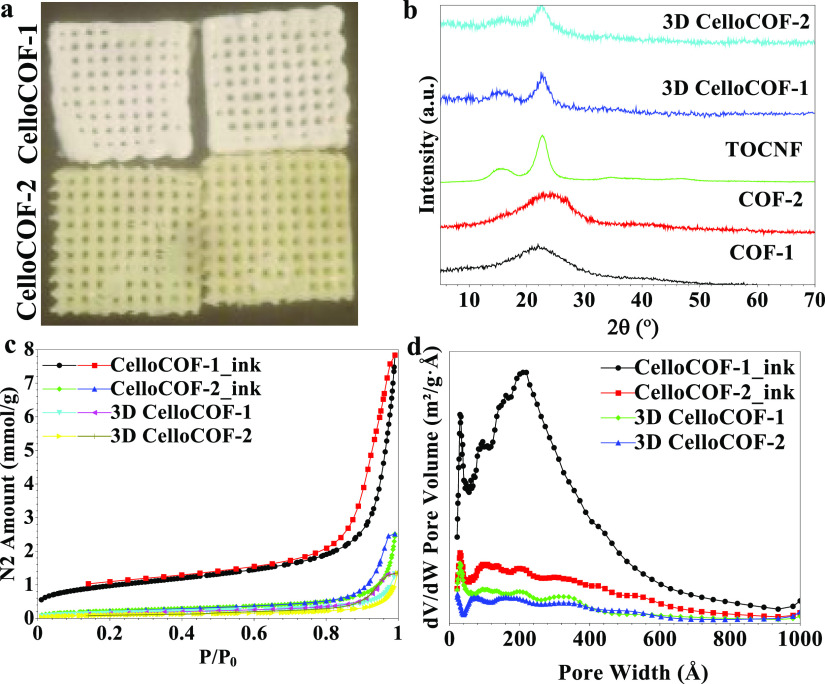
(a) Camera photos for 3D CelloCOF-1 using different pore structures and characterization of COF and 3D CelloCOF using (b) XRD, (c) nitrogen adsorption–desorption isotherms, and (d) PSD.

The methodology for the fabrication of COF-1 and COF-2 inks for utilization in 3D printing is illustrated in Figure 1. COF-1 and COF-2 are synthesized by using the polycondensation reaction involving melamine and either terephthalaldehyde or benzene-1,3,5-tricarboxyaldehyde, as depicted in Figure 1. The ink preparation involves the combination of SA and TOCNF. The process of fabricating cubes using the 3D printing technology with a pore size of 1 mm was conducted using various dimensions, as seen in Figure 2a.

XRD patterns for COF-1, COF-2, TOCNF, 3D CelloCOF-1, and 3D CelloCOF-2 are listed in Figure 2b. COF-1 and COF-2 display broad peaks at Bragg angles of 22 and 23°, respectively, indicating the low crystallinity of the materials or the small particle size causing peak broadening according to Scherrer’s equation (Figure 2b). The XRD pattern for TOCNF shows three diffraction peaks at 15.6, 16.7, and 22.7° that can be assigned to Miller indices of (1ı̅0), (110), and (200), respectively, for cellulose I_β_.^43^ The diffraction peaks can be assigned to (100), (010), and (110) for the counterparts of the cellulose I_α_.^43^ The average crystallite size corresponding to the fiber’s width was calculated by using Scherrer’s equation. The analysis using the (200) diffraction peak at Bragg angle 22.7° indicates a width size of 3.89 nm (Figure 2b). The 3D CelloCOF-1 and 3D CelloCOF-2 display the main characteristic diffraction peaks for the TOCNF and COF materials (Figure 2b).

The porosity of the inks and 3D printed scaffolds is evaluated by using nitrogen sorption isotherms (Figure 2c). Data analysis shows BET-specific surface areas of 128, 39, 30, and 11 m^2^/g for CelloCOF-1 ink, CelloCOF-2 ink, 3D CelloCOF-1, and 3D CelloCOF-2, respectively. The PSD of the inks and 3D printed CelloCOF is shown in Figure 2d. Data analysis shows maximum volume at pore sizes of 3.1 and 20.4 nm for all materials. It is also important to note that the 3D printed scaffolds show a custom pore of 1 mm (Figure 2a).

**Figure 3:**
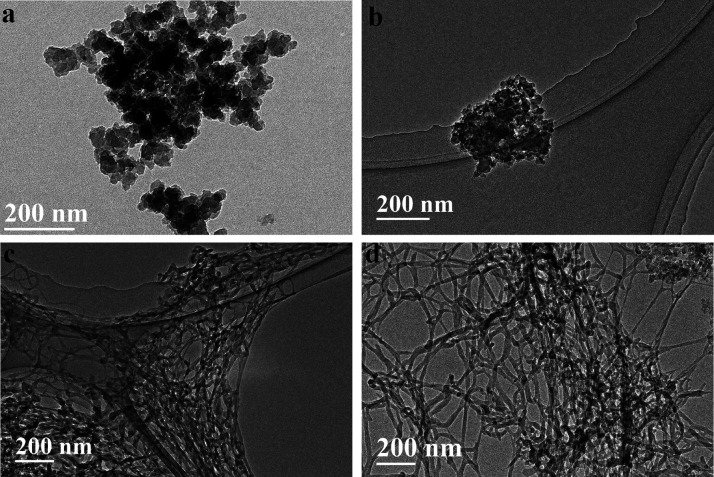
TEM images for (a) COF-1, (b) COF-2, (c) COF-1/TOCNF, and (d) COF-2/TOCNF.

The TEM images for the COFs and the COFs/TOCNF are recorded in Figure 3. COF materials display good dispersion inside the entangled TOCNF nanofibrils (diameter of 1–3 nm). COF-1 and COF-2 show particle sizes of 20–25 nm (Figure 3a,b). They tend to aggregate, forming large particles. The aggregation of COF materials decreases the material performance and blocks the materials’ intrinsic porosity. The dispersion of COF particles into the TOCNF prevents the aggregation of COF materials (Figure 3c,d).

**Figure 4:**
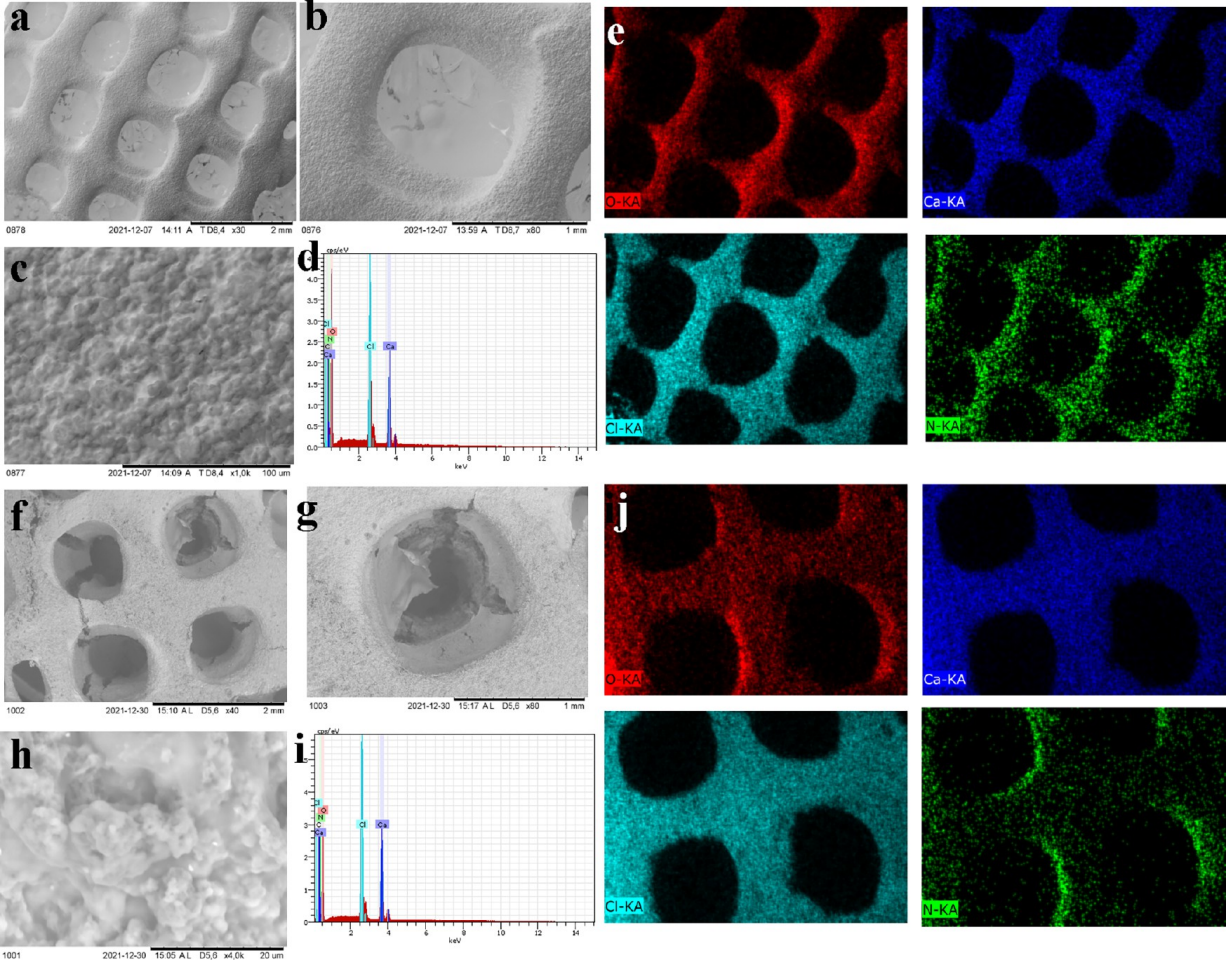
(a,b,c, f, g,h) SEM image, (d, i) EDX analysis, and (e, j) mapping for (a–e) 3D CelloCOF-1 and (f–j) 3D CelloCOF-2.

The 3D CelloCOFs were imaged by using SEM (Figure 4). They show the pore size of 1 mm that was custom-made using a CAD model during 3D printing (Figure 4). The printed scaffolds exhibit a well-organized pore structure offering hierarchical porous materials containing a macropore (1 mm) regime formed between the CelloCOF crystals and a micropore regime due to the COF crystal. EDX analysis confirmed the presence of elements C, N, O, Ca, and Cl (Figure 4d–i). No extra elements were observed, indicating that the printing objects contain high purity of the prepared scaffolds with only TOCNF, COFs, and sodium alginate cross-linked CaCl_2_. The elemental distribution of the N element of COF materials shows the homogeneous distribution of COFs inside the 3D printed objects (Figure 4e–j).

3D printing of COF using TOCNF/SA offered several advantages. The TOCNF/SA blend is cheap compared to gelatin/sodium alginate.^7^ Cellulose/SA is biocomptabile compared to surfactants, e.g., Pluronic F127.^10^ Our method offers a DIW procedure. TOCNF/SA enabled coassembly with COFs in an aqueous solution. There is no need for a 3D printed template or heating.^10^ There is no observation for cracking compared to the reported method using PF-127, cellulose acetate, or mixed cellulose ester (containing cellulose acetate and cellulose nitrate).^8^ These observations reveal the potential of our method compared to the previously reported procedures.^7,8,10^

### Application for CO_2_ Sorption

**Figure 5:**
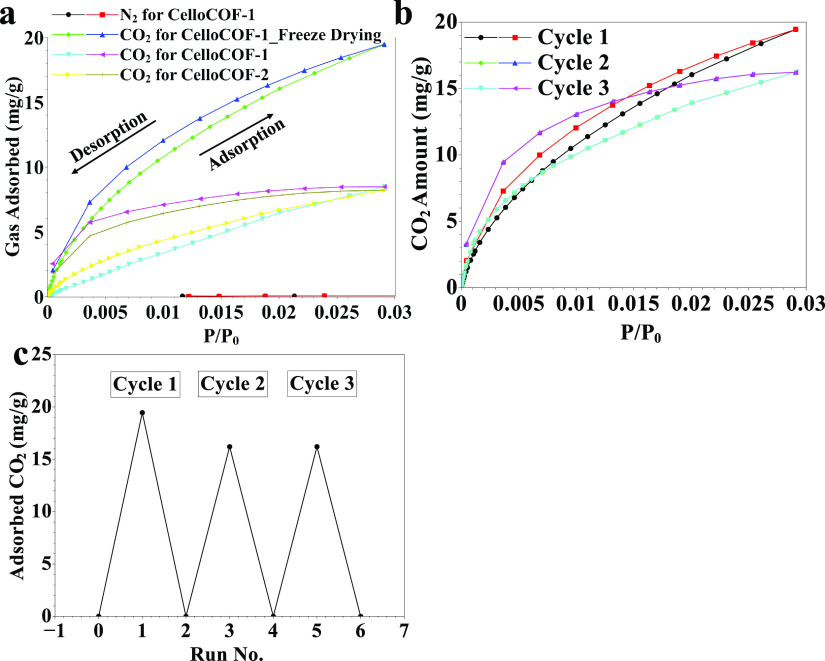
(a) CO_2_ adsorption and (b, c) recyclability for CelloCOF-1.

The adsorption–desorption isotherms of CO_2_ using 3D CelloCOFs materials are investigated (Figure 5). 3D CelloCOF-1 dried via freeze-drying, 3D CelloCOF-1, and 3D CelloCOF-2 exhibit adsorption capacities of 19.9, 8.5, and 8.2 mg/g, respectively (Figure 5a). Freeze-drying enabled a 2.3-fold increase in the adsorption performance for CO_2_ with good selectivity over that of nitrogen gas (Figure 5a). The adsorption and desorption processes are reversible (Figure 5b). Thus, the material can be used several times without any decrease in performance (Figure 5c). The CO_2_ adsorption decreased from 19.5 to 16.2 mg/g, representing only a 16.9% decrease in the adsorption capacity (Figure 5c). The CO_2_ adsorption takes place through pressure-swing adsorption (PSA) and vacuum-swing adsorption (VSA) processes.^21^

Our 3D printed COF offered a CO_2_ adsorption of 19.5 mg/g, which is comparable to other 3D printed Schiff-base networks (SNW-1, CO_2_ uptakes of 58.42 and 41.41 cm^3^/g at 273 and 298 K, respectively).^8^ A Schiff-base COF (TpPa-1) of 2,4,6-triformyl phloroglucinol (Tp) and 1,4-diaminobenzene (Pa) was reported for CO_2_ capture.^44^ TpPa-1 exhibited keto-enamine moieties with a CO_2_ capacity of 0.6 mmol/g and a CO_2_/N_2_ sorption selectivity of 114.^44^ 3D CelloCOF exhibits a high adsorption capacity with a good selectivity of 491 (Figure 5a).

### PFOS Adsorption

**Figure 6:**
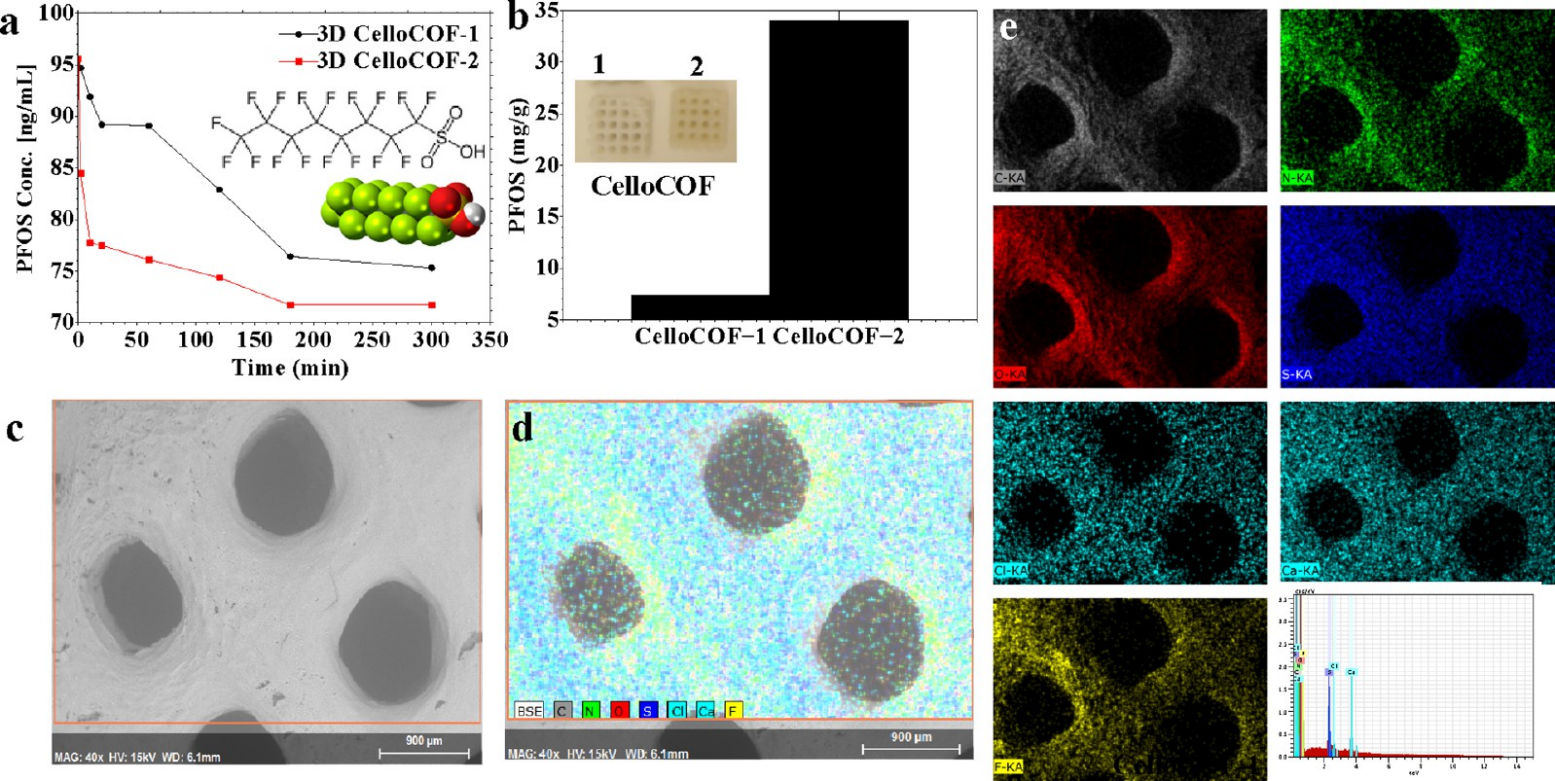
Adsorption of PFOS using CelloCOF. (a) Kinetic study for the adsorption process. The inset presents the chemical structure of PFOS molecules. (b) Adsorption capacity. The inset presents the printed materials after adsorption. (c) SEM and (d, e) EDX analysis/mapping for CelloCOF-2 after adsorption.

The adsorption of PFOS was evaluated by using 3D CelloCOFs, as shown in Figure 6. The kinetics of the PFOS adsorption indicate fast adsorption within the first 20 min (Figure 6a). 3D CelloCOF-1 and 3D CelloCOF-2 exhibit similar behaviors, showing an equilibrium at 180 min (Figure 6a). However, we tested the adsorption for 12 h to determine the adsorption capacity using an initial concentration of 1000 ppm (Figure 6b). 3D CelloCOF-1 and three-dimensional CelloCOF-2 offer adsorption capacities of 7.4 and 34 mg/g, respectively (Figure 6b). The inset of Figure 6b for the material after adsorption indicates that the materials preserve their morphology, structure, and dimensional stability after adsorption.

**Figure 7:**
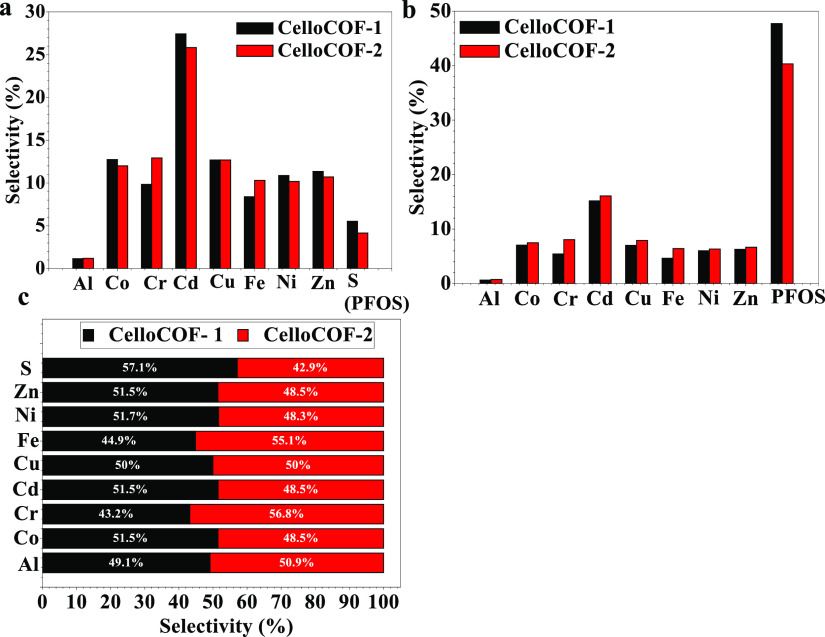
Selectivity of PFOS (reported measuring (a) ‘S’ and (b) PFOS) in the presence of heavy metals and (c) comparable selectivity of each element for CelloCOF-1 and CelloCOF-2.

The adsorption of PFOS on the 3D CelloCOFs is evaluated using EDX analysis and mapping (Figure 6c–e). The SEM image of PFOS@3D CelloCOF-2 shows a precipitate on the external surface of the materials (Figure 6c). The elemental analysis of the observed precipitate using EDX mapping (Figure 6d) and analysis (Figure 6e) confirms the presence of PFOS molecules. EDX analysis and mapping confirm the presence of S and F elements belonging to PFOS molecules (Figure 6d,e). EDX mapping of these elements indicates the homogeneous distribution of PFOS on the surface of 3D CelloCOF-2 (Figure 6d,e). The selectivity of PFOS in the presence of other interferences such as heavy metals is tested as shown in Figure 7a. In a mixture of heavy metals and PFOS, cadmium ions exhibit a high selectivity of 27% (Figure 7a). The materials exhibit the highest selectivity of 5% toward PFOS in terms of sulfur. If we consider the molecular weight of PFOS (500 g/mol), this selectivity can reach 47.4 and 40.3% for CelloCOF-1 and CelloCOF-2, respectively (Figure 7b). CelloCOF-1 and CelloCOF-2 display similar adsorption behavior in the presence of heavy metal ions due to the similar chemical structure of both adsorbents (Figure 7c).

The structure of the COF materials displays an important role in the adsorption performance of PFOS molecules. 3D CelloCOF-2 exhibits higher adsorption capacity than 3D CelloCOF-1 (Figure 6b). The high adsorption performance for 3D CelloCOF-2 is due to the pore size of COF-2, which offers a large pore size compared to COF-1. It shows the homogeneous distribution of the PFOS molecules on the external surface of the printed materials (Figure 6d,e). In contrast, the adsorption of PFOS on 3D CelloCOF-1 is heterogeneously distributed (Figure S1). EDX analysis and mapping of PFOS-adsorbed 3D CelloCOF-1 indicate a small adsorption amount of the molecules on the surface of the adsorbent. Data show also that the adsorbed molecules are localized surrounding the pore of the printed object (Figure S1). The interaction between PFOS and 3D CelloCOF has been evaluated by using FT-IR spectra (Figure S2). There is no dramatic change in the spectrum of 3D CelloCOF after the adsorption of PFOS, indicating that the process is mainly due to noncovalent interactions (Figure S2).

A summary of the material’s application for PFOS adsorption is given in Table 1. Adsorbents, such as zeolite,^45,46^ metal–organic frameworks (MOFs),^47,48^ reduced graphene oxide (rGO)-modified zinc ferrite (ZF) immobilized chitosan beads (CB) (denoted as rGO-ZF@CB),^49^ and aluminum-based water treatment residuals (Al-WTR),^50^ were reported for PFOS adsorption (Table 1). 3D CelloCOF exhibits high adsorption capacity within a short equilibrium time compared to other materials (Table 1). COF powder is difficult to separate from an aqueous solution. Thus, magnetic covalent triazine frameworks (CTF/Fe_3_O_4_) were synthesized via the ball-milling method.^25^ CTF/Fe_3_O_4_ was applied to separate sodium *p*-perfluorous nonenoxybenzenesulfonate (OBS) and hexafluoropropylene oxide trimer acid (HFPO-TA). It offered adsorption capacities of 1.18 and 1.02 mmol/g for HFPO-TA, respectively.^25^ Our materials can be easily separated from the aqueous solution without centrifugation or an external magnet.

**Table 1:** Adsorption of PFOS Using Different Materials^a^

**type**	**materials**	**form**	**capacity (mg**/**g)**	**time (h)**	**ref**
zeolite	Fe-BEA35	powder	19.6	96	(45)
	NaY		12	4	(46)
MOFs	SCU-8		44.79	0.03	(47)
	MIL-125-NH_2_		17		(48)
carbon/metal oxide/chitosan	rGO-ZF@CB	beads	21.64	2	(49)
waste	Al-WTR	powder	0.316	24	(50)
3D CelloCOF	cellulose-COFs	3D printed	7.4–34	3	this study

a SCU-8, Soochow University; MIL-125-NH_2_, Materials Institute Lavoisier.

### Heavy Metal Adsorption

**Figure 8:**
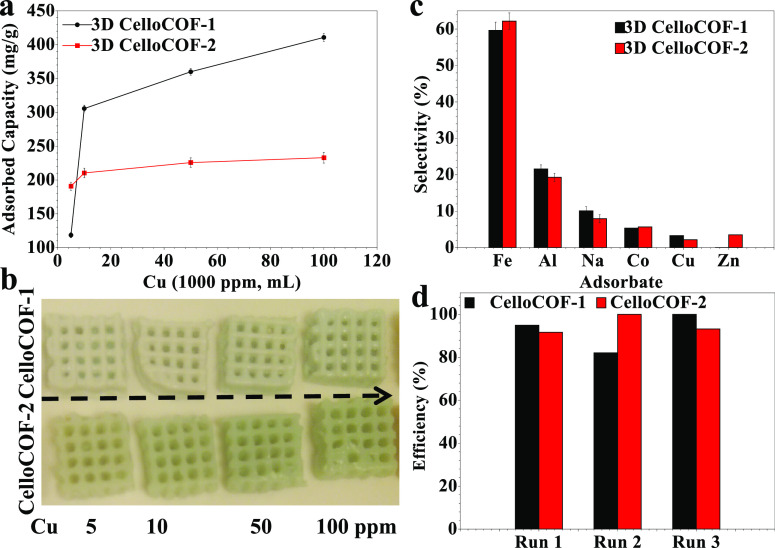
Adsorption of Cu^2+^ ions using CelloCOF: (a) adsorption capacity, (b) CelloCOF after Cu^2+^ ion adsorption, (c) selectivity, and (d) recyclability toward copper ion adsorption.

The metal adsorption of Cu^2+^ ions using 3D CelloCOFs is evaluated (Figure 8a,b). The adsorption increases with the increase in the initial concentration of Cu^2+^ ions (Figure 8a). 3D CelloCOF-1 exhibits a higher adsorption capacity than 3D CelloCOF-2 (Figure 8a). It shows adsorption capacities of 118.5 305.6, 360, and 410.8 mg/g for Cu^2+^ ions (1000 ppm) volumes of 5, 10, 50, and 100 ppm, respectively (Figure 8a). On the other side, 3D CelloCOF-1 exhibits adsorption capacities of 190.8 210.4, 225.7, and 233 mg/g, respectively (Figure 8a). The increased adsorption of Cu^2+^ ions can be easily evaluated by using the naked eye (Figure 8b). There is no damage or change in the pore structure of the printed materials after metal ion adsorption (Figure 8b).

The selectivity for heavy metal adsorption is determined using metals of Fe^3+^, Al^3+^, Co^2+^, Cu^2+^, Zn^2+^, and Na^+^ (Figure 8c). 3D CelloCOF-1 shows selectivities (%) of 59.6, 21.6, 10.1, 5.3, 3.3, and 0% for Fe^3+^, Al^3+^, Na^+^, Co^2+^, Cu^2+^, and Zn^2+^, respectively. On the other side, 3D CelloCOF-2 shows selectivities (%) of 62.2, 19.3, 7.9, 5.7, 2.2, and 3.2% for Fe^3+^, Al^3+^, Na^+^, Co^2+^, Cu^2+^, and Zn^2+^, respectively (Figure 8c). The yellow color of the materials can confirm the high selectivity toward Fe^3+^ ions after adsorption. No damage or change in the material’s morphology or structure indicates the high stability of the printed objects to use for mixed metal ions and industrial wastewater. Thus, the materials can be used for several recyclings without a significant decrease in the adsorption efficiency for Cu^2+^ ions as a model (Figure 8d).

**Figure 9:**
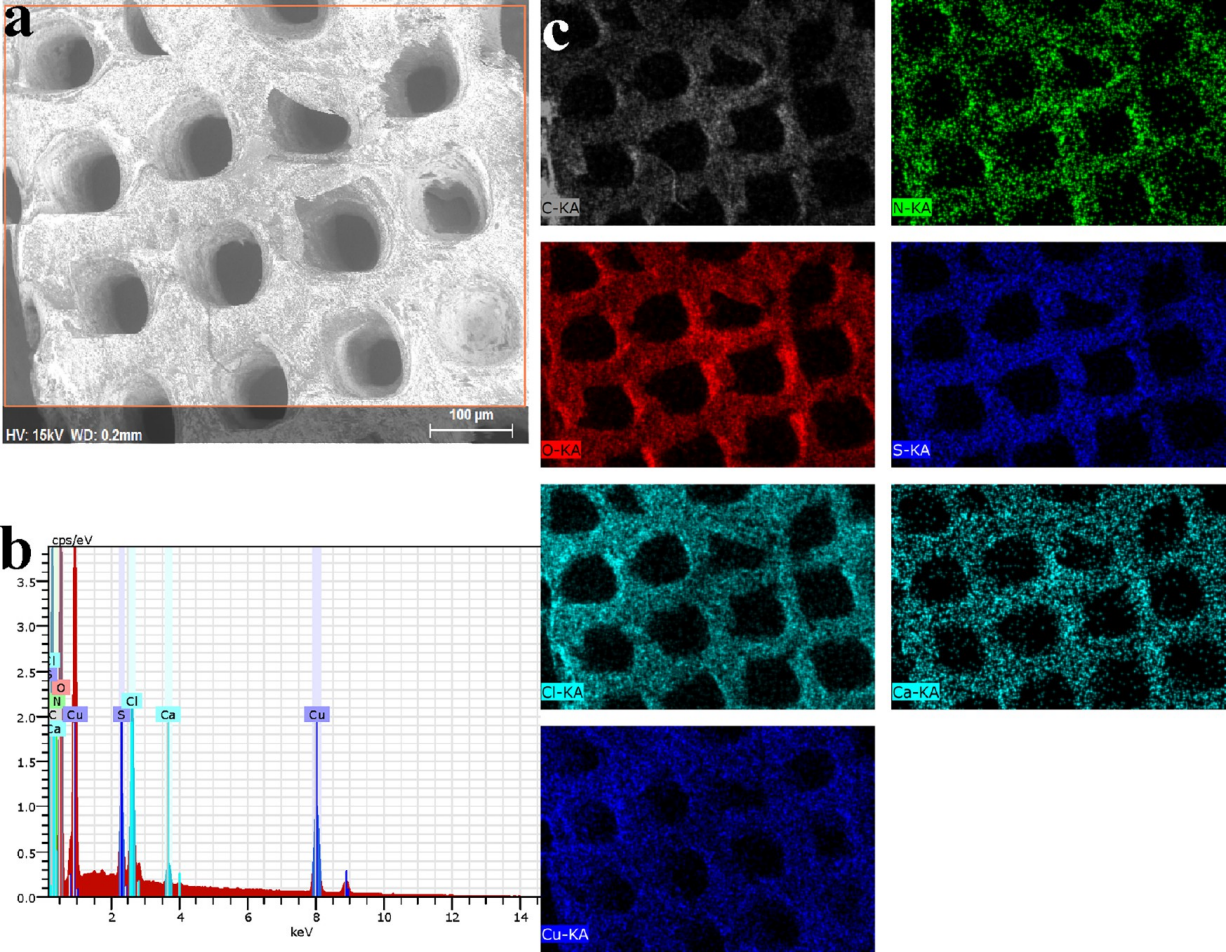
CelloCOF-2 after adsorption of Cu^2+^ ions: (a) SEM image, (b) EDX analysis, and (c) EDX mapping.

The adsorption of heavy metal ions such as Cu^2+^ on 3D CelloCOF-1 (Figure S3) and 3D CelloCOF-2 (Figure 9) is characterized by using SEM, EDX analysis, and mapping. The SEM images show white contrast particles due to Cu^2+^ ions that can be confirmed by EDX analysis. The adsorbed Cu^2+^ ions are homogeneously distributed on the adsorbent CelloCOFs (Figure 9). The interaction inside Cu-adsorbed CelloCOF-2 was evaluated by using FT-IR spectra (Figure S4). The changes in the N-based functional groups, e.g., N–H and C=N, indicate that the adsorbed Cu^2+^ ions have interacted with these functional groups via coordination with the unpaired electron or nitrogen atoms inside the COF crystals. On the other side, the adsorption of Fe^3+^ ions is placed on the functional groups of TOCNF, i.e., O–H or COO^–^. The TGA curves of Cu-adsorbed CelloCOF-1 (Figure S5) and CelloCOF-2 (Figure S6) were recorded. The residual at a temperature higher than 500 °C represents CuO that comes from the adsorbed species. The TEM images of the residuals indicate the presence of CuO embedded in a gray layer of carbon (Figure S7). The adsorbed metals-CelloCOFs or the materials after carbonization could be effective for further applications as catalysts.

Ethylenediaminetetraacetic acid (EDTA)-functionalized TpPa-NH_2_ COF (TpPa-NH_2_@EDTA) was reported to remove heavy metal ions (Table 2).^51^ Azine-linked COF (ACOF) of 1,3,5-triformyl phloroglucinol (TFP) and hydrazine was prepared via solvothermal synthesis (at 80 °C for 3 days) for heavy metal adsorption.^52^ The materials require a long equilibrium time (Table 2). A triarylamine-based COF (TNPP) was reported for the heavy metal removal of mercury (Hg^2+^) ions selectively with a capacity of 95 mg/g compared to other metal ions due to the presence of thiol functional groups.^53^ 2D COF containing TFP and oxalyldihydrazide (ODH), denoted as TpODH, was synthesized via hydrazone bonds that facilitate the heavy metal adsorption.^54^ The presence of functional groups, such as sulfhydryl-functionalized COF (SH), improved the selectivity toward certain heavy metal adsorption, e.g., Pb^2+^ ions.^55^ However, it requires a very long time for equilibrium and limits the material’s performance in removing other pollutants (Table 2).

**Table 2:** Heavy Metal Adsorption Using a COF-Based Adsorbent^a^

**COFs**	**form**	**heavy metals**	**capacity** (mg/g)	**time**	**ref**
TpPa-NH_2_@EDTA	powder	Ag^+^, Pd^2+^, Fe^3+^, Cr^3+^, Cu^2+^, and Ni^2+^	232.55	10 min	(51)
ACOF		U^6+^ and Hg^2+^	169–175	5 min	(52)
TNPP		Cr^3+^, K^+^, Ag^+^, Ca^2+^, Co^2+^, Pb^2+^, Cu^2+^, Cd^2+^, Fe^3+^, Ba^2+^, and Na^+^	95	NA	(53)
TpODH		Hg^2+^, Cu^2+^, Pb^2+^, Cr^3+^, and Cd^2+^	324	250 min	(54)
COF-SH		Cu^2+^, Fe^3+^, Cd^2+^, Mn^2+^, Cr^6+^, and Pb^2+^	225.7	48 h	(55)
3D CelloCOF	scaffold	Cu^2+^, Co^2+^, Zn^2+^, Ca^2+^, Fe^3+^, Al^3+^, and Na^+^	118.5–410.8	180 min	this study

a NA, not applicable.

## Conclusions

In this study, we present a comprehensive approach for the 3D printing of CelloCOF using the DIW technique. The approach employed in our study facilitated the creation of cellulose/alginate/COF scaffolds without the need for complex protocols or individually tailored 3D macrostructures. The process enabled the uniform incorporation of COFs into cellulose/alginate matrices, resulting in well-connected interfaces at the molecular scale. The utilization of 3D CelloCOF as an adsorbent has been explored for its potential to capture CO_2_, heavy metals, and PFOS. These materials demonstrate a notable capacity for adsorption, along with favorable characteristics in terms of recyclability and selectivity. The straightforward printing methodology and the commendable adsorption efficacy, encompassing a wide range of pollutant applicabilities and substantial adsorption capabilities, present auspicious materials for use in industrial settings.

## Supporting Information Available

Scanning electron microscopy (SEM) images and energy-dispersive analysis (EDX) and mapping for CelloCOF after adsorption of PFOS and Cu^2+^ ions, FT-IR spectra for CelloCOF before and after adsorption of PFOS and heavy metal ions, TGA curves for CelloCOFs after adsorption of Cu^2+^ ions, TEM images for TGA residuals of CelloCOF after adsorption of Cu^2+^ ion and mixed metal ions (PDF)
