# Supplementary material for: Three-Dimensional Printing of Cellulose/Covalent Organic Frameworks (CelloCOFs) for CO2 Adsorption and Water Treatment
Source: ACS Appl Mater Interfaces. 2023 Dec 14;15(51):59795–805. doi: 10.1021/acsami.3c13966 (PMC10755704; doi:10.1021/acsami.3c13966)
Supplement: Supplementary file 1 — am3c13966_si_001.pdf [file am3c13966_si_001.pdf]

Supporting Information

Three-dimensional (3D) Printing of Cellulose/Covalent Organic Frameworks (CelloCOFs) for CO_2 Adsorption and Water Treatment

Hani Nasser Abdelhamid[1,2,3]*, Sahar Sultan[1], Aji P. Mathew[1]*

[1]Division of Materials and Environmental Chemistry, Stockholm University, Svante Arrhenius väg 16 C, Stockholm, SE-10691, Sweden
[2]Advanced Multifunctional Materials Laboratory, Department of Chemistry, Faculty of Science, Assiut University, Assiut, 71515, Egypt
[3]Nanotechnology Research Centre (NTRC), The British University in Egypt (BUE), El-Shorouk City, Suez Desert Road, P.O. Box 43, Cairo 11837, Egypt
*Corresponding to Abdelhamid (hany.abdelhamid@aun.edu.eg); Mathew (aji.mathew@mmk.su.se)

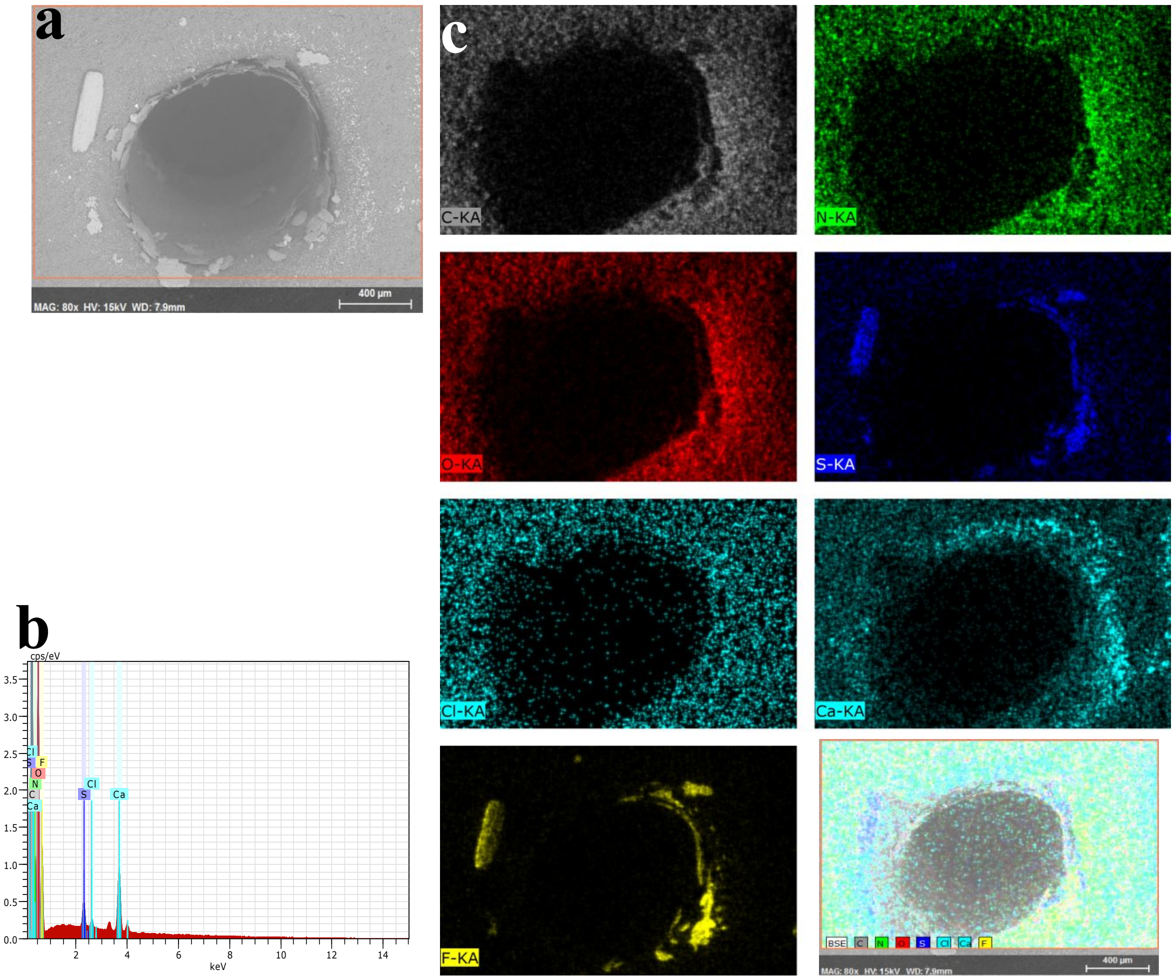

Figure S1 a) SEM image, b) EDX analysis, and c) EDX mapping for CelloCOF-1 after adsorption of PFOS.

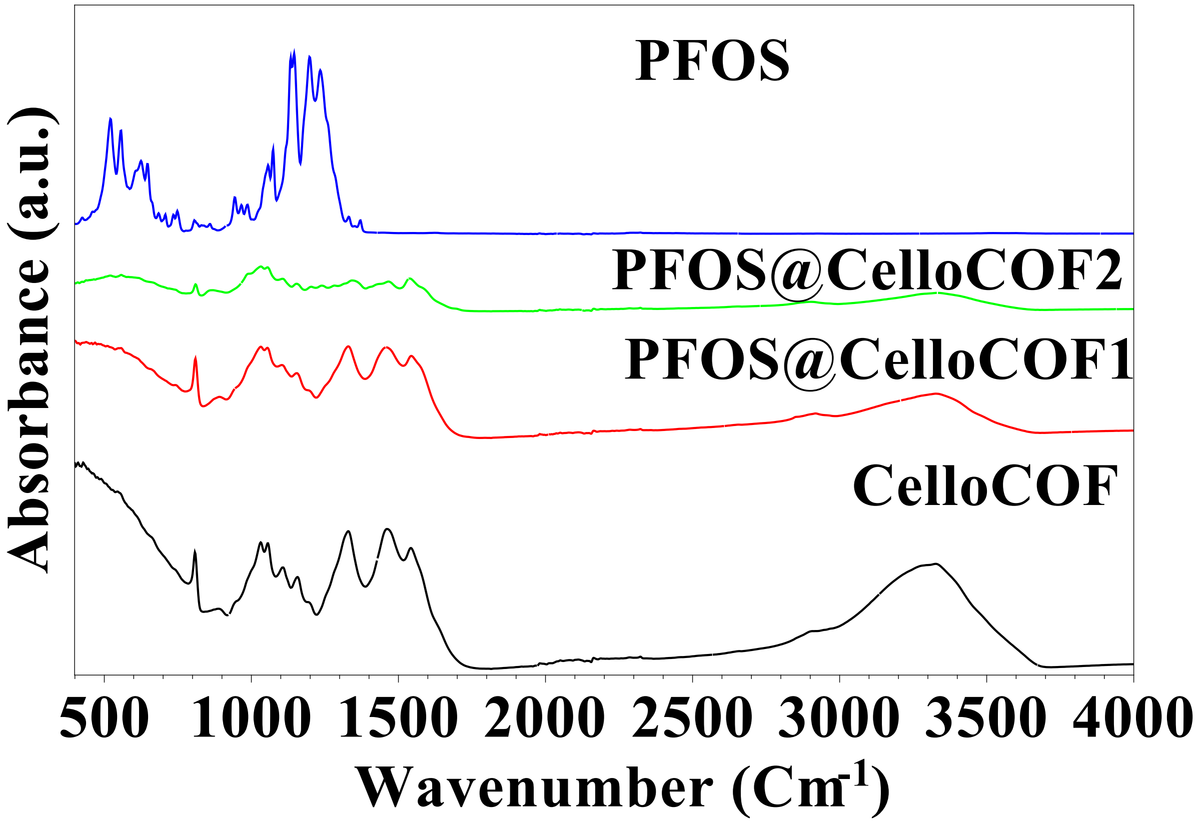

Figure S2 FT-IR spectra for CelloCOF before and after adsorption of PFOS.

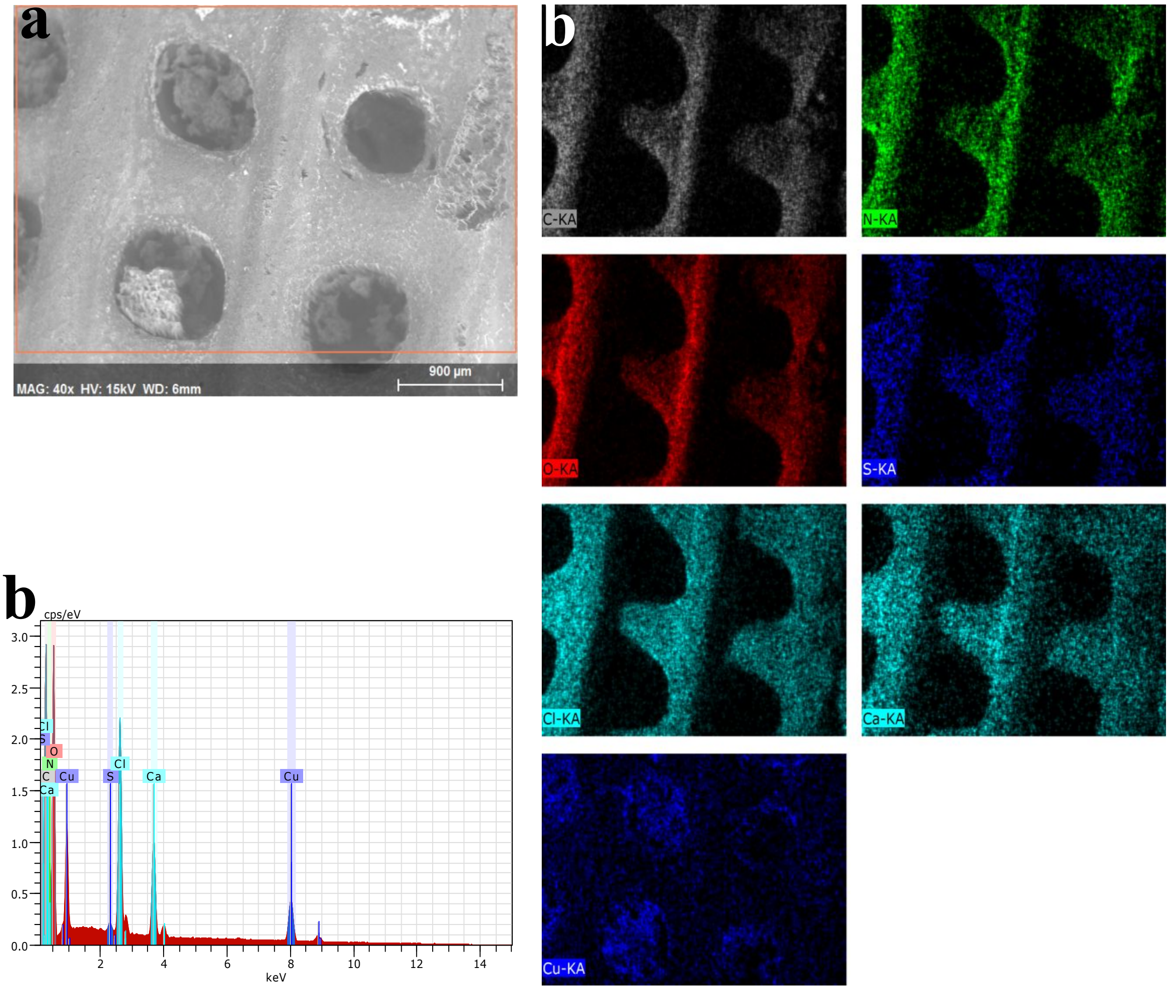

Figure S3 a) SEM image, b) EDX analysis, and c) EDX mapping for CelloCOF-1 after adsorption of Cu^{2+} ions.

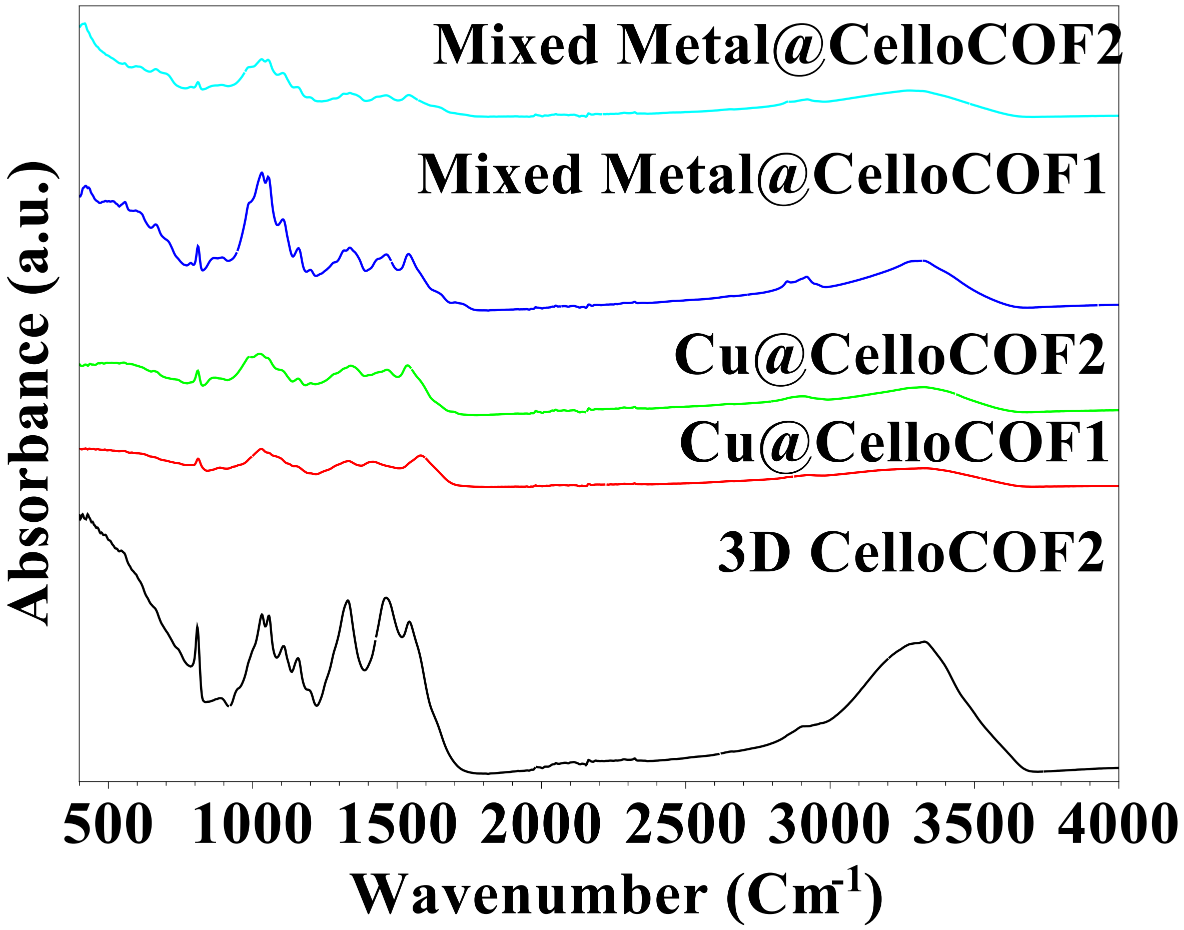

Figure S4 FT-IR spectra for CelloCOF before and after adsorption of heavy metal ions.

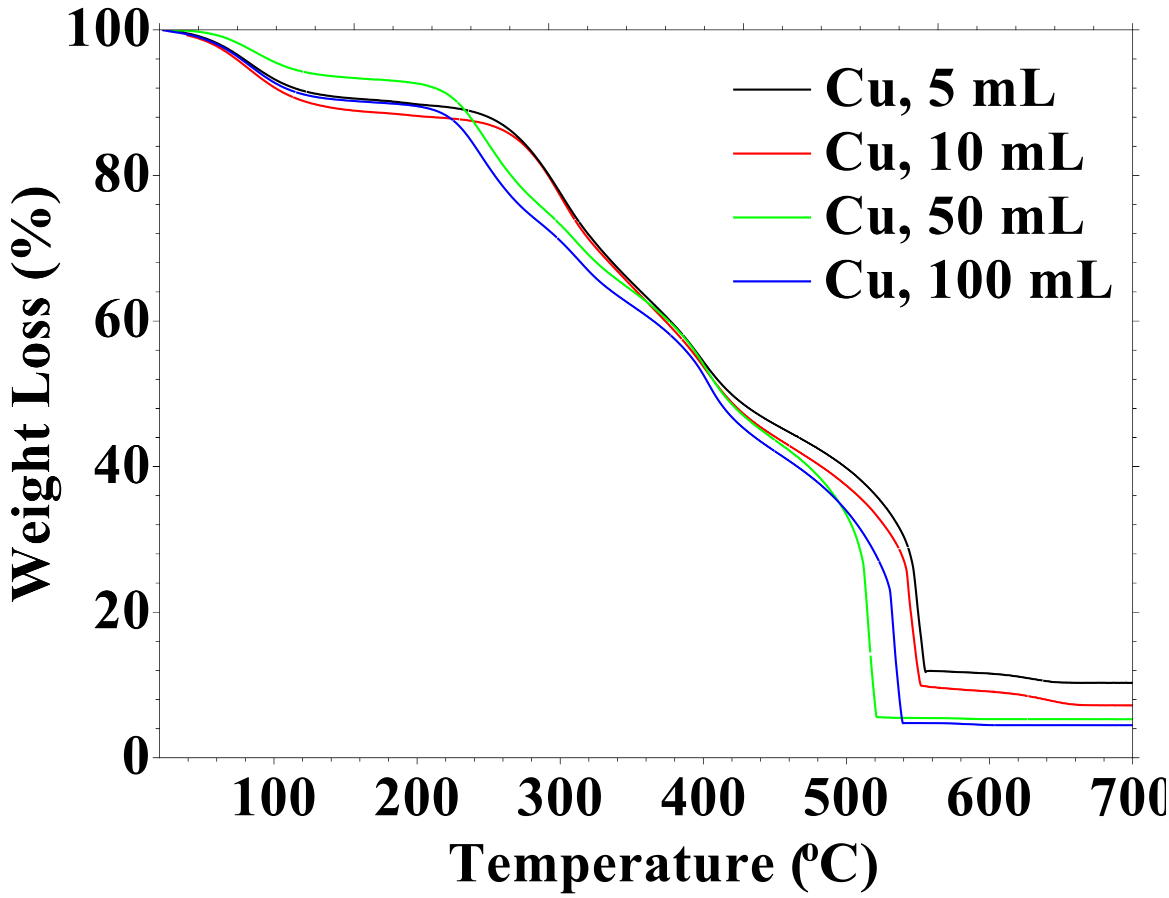

Figure S5 TGA for CelloCOF-1 after adsorption of Cu^{2+} ions.

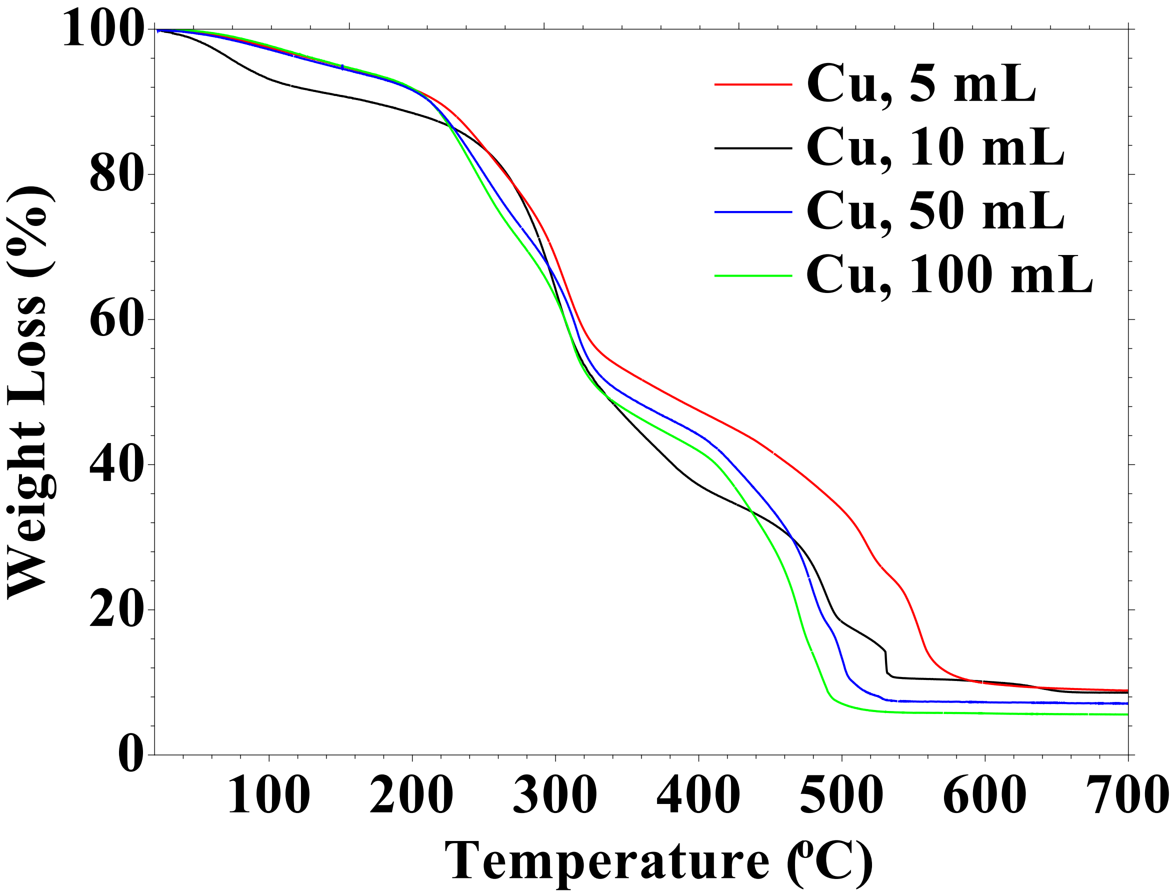

Figure S6 TGA for CelloCOF-2 after adsorption of Cu^{2+} ions.

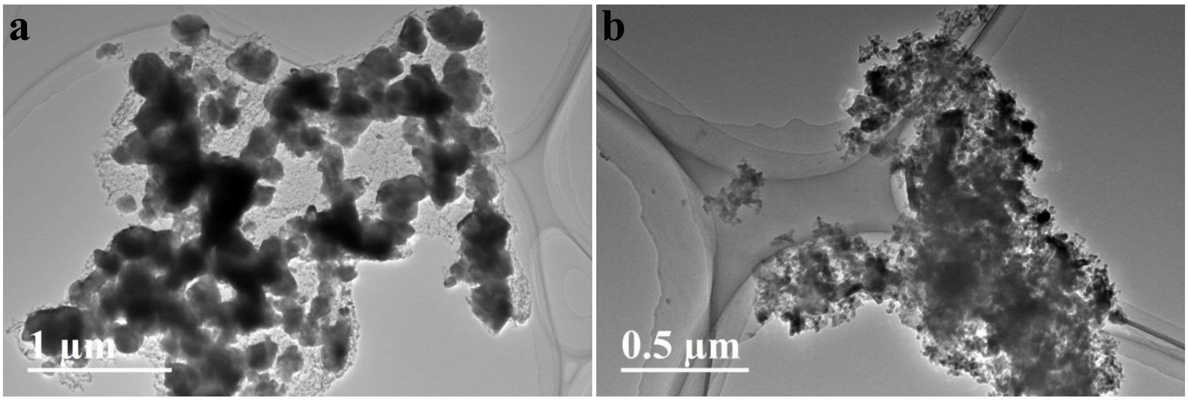

Figure S7 TEM images for TGA residuals of CelloCOF after adsorption of Cu^{2+} ion, and b) mixed metal ions.
